# Supplementary material for: Differential spatial computations in ventral and lateral face-selective regions are scaffolded by structural connections
Source: Nat Commun. 2021 Apr 15;12:2278. doi: 10.1038/s41467-021-22524-2 (PMC8050273; doi:10.1038/s41467-021-22524-2)
Supplement: Supplementary file 1 — Supplementary Information [file 41467_2021_22524_MOESM1_ESM.pdf]

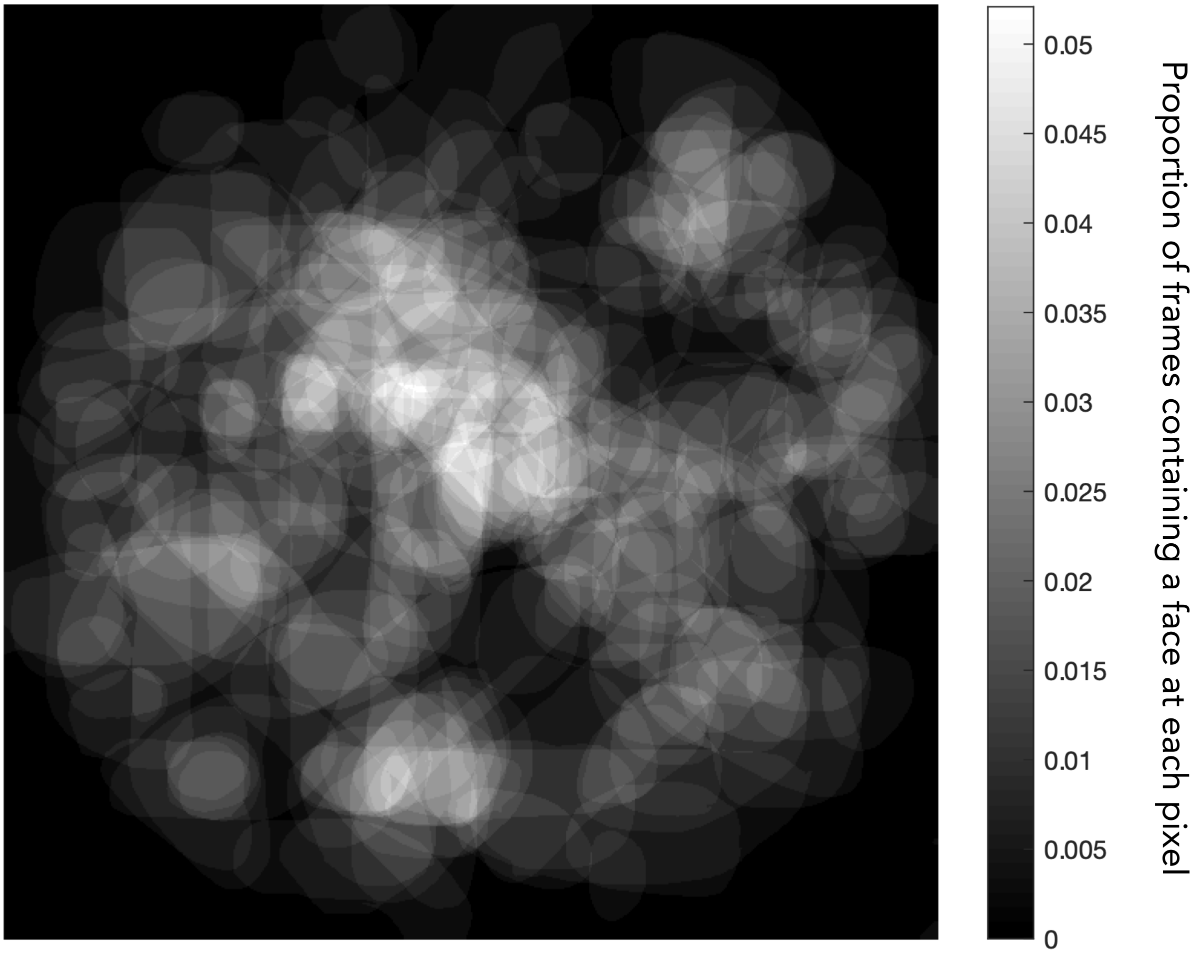

Supplementary Fig. 1: Location of face (vs. non-face) stimuli in the "toonotopy" experiment. To quantify the location of face stimuli across the visual field in our pRF mapping experiment, we created a binary mask for each frame of the experiment, where white indicated a face stimulus within the aperture and black indicated a non-face stimulus or background. Averaging these binary masks together, we see that face stimuli fairly comprehensively tile the circular aperture and are not solely located in the center of the display. To quantify this, we divided the image based on corresponding degrees of eccentricity when displayed to the subject and grouped pixels according to the four eccentricity bands used for analysis throughout the paper (0°–5°; 5°–10°; 10°–20°; 20°–40°). The proportion of frames containing face stimuli averaged across pixels in each eccentricity band were 0.0367, 0.0292, 0.0202, and 0.0128 for the 0°–5°, 5°–10°, 10°–20° and 20°–40° bands, respectively. This illustrates that while the proportion of faces is highest in the center of the display and decreases as we approach the far periphery, there are still a substantial number of frames including face stimuli presented out to 40°.

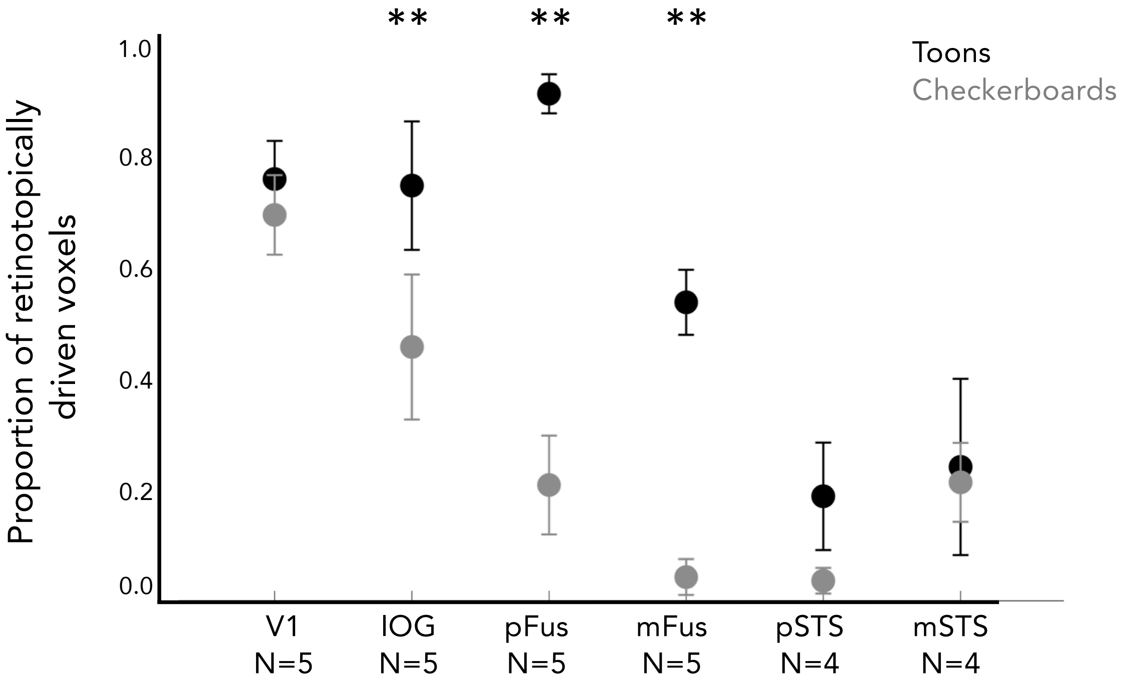

Supplementary Fig. 2: Proportion of left hemisphere voxels in each region that the pRF model explains more than 20% of their variance. Data are averaged across five participants who underwent both checkerboard retinotopy and toonotopy. *Error bars:* standard error of the mean. Asterisks indicate significant increases in the proportion of retinotopically modulated voxels for toonotopy vs. standard checkerboard retinotopy as measured by post-hoc Tukey t-tests (** indicates $p < .01$, two-sided). IOG-faces: $t(40)=2.75$, $p=.0089$, $d=0.43$; pFus-faces: $t(40)=6.66$, $p=5.50\times10^{-8}$, $d=1.05$; mFus-faces: $t(40)=4.68$, $p=3.27\times10^{-5}$, $d=0.74$; pSTS-faces: $t(40)=1.29$, $p=.21$; mSTS-faces: $t(40)=0.24$, $p=.82$; V1: $t(40)=0.61$, $p=.54$. Corresponding main text figure: figure 1C. Source data are provided as a Source Data file.

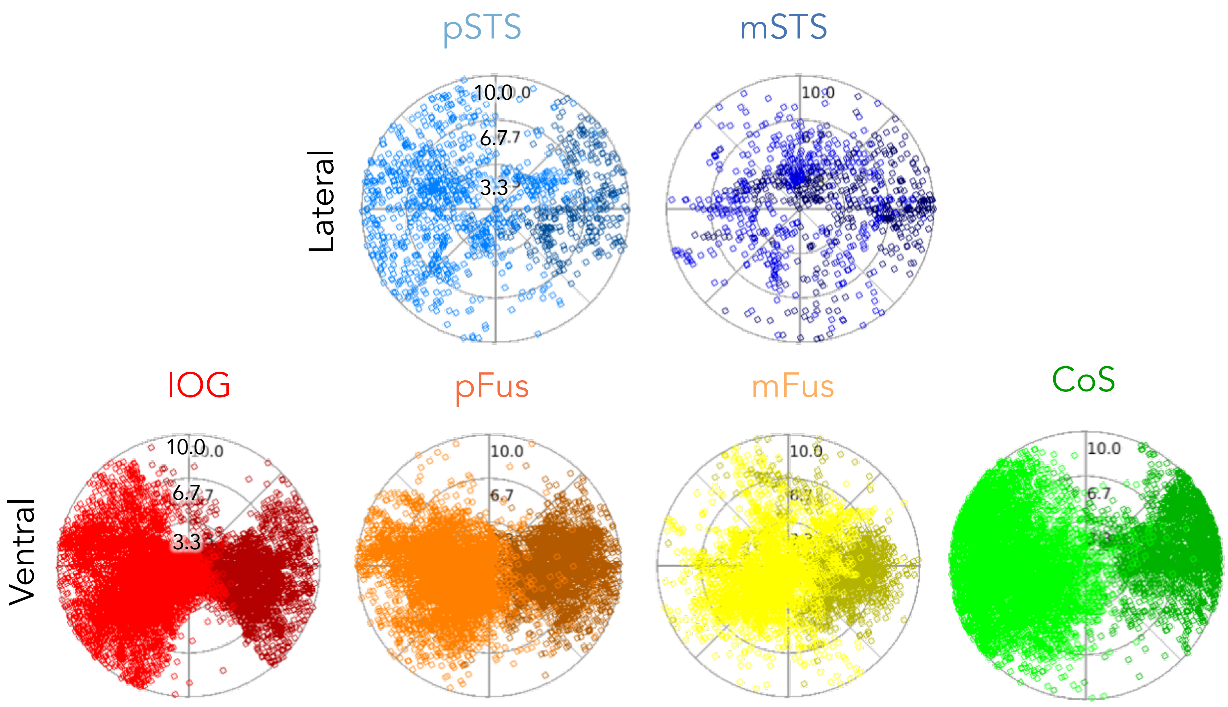

Supplementary Fig. 3: Distribution of pRF centers for the central 10 degrees
Each dot is a pRF center. Data are shown for all pRFs across all participants for both hemispheres. *Dark colors:* Left hemisphere. Corresponding main text figure: figure 2A. Source data are provided as a Source Data file.

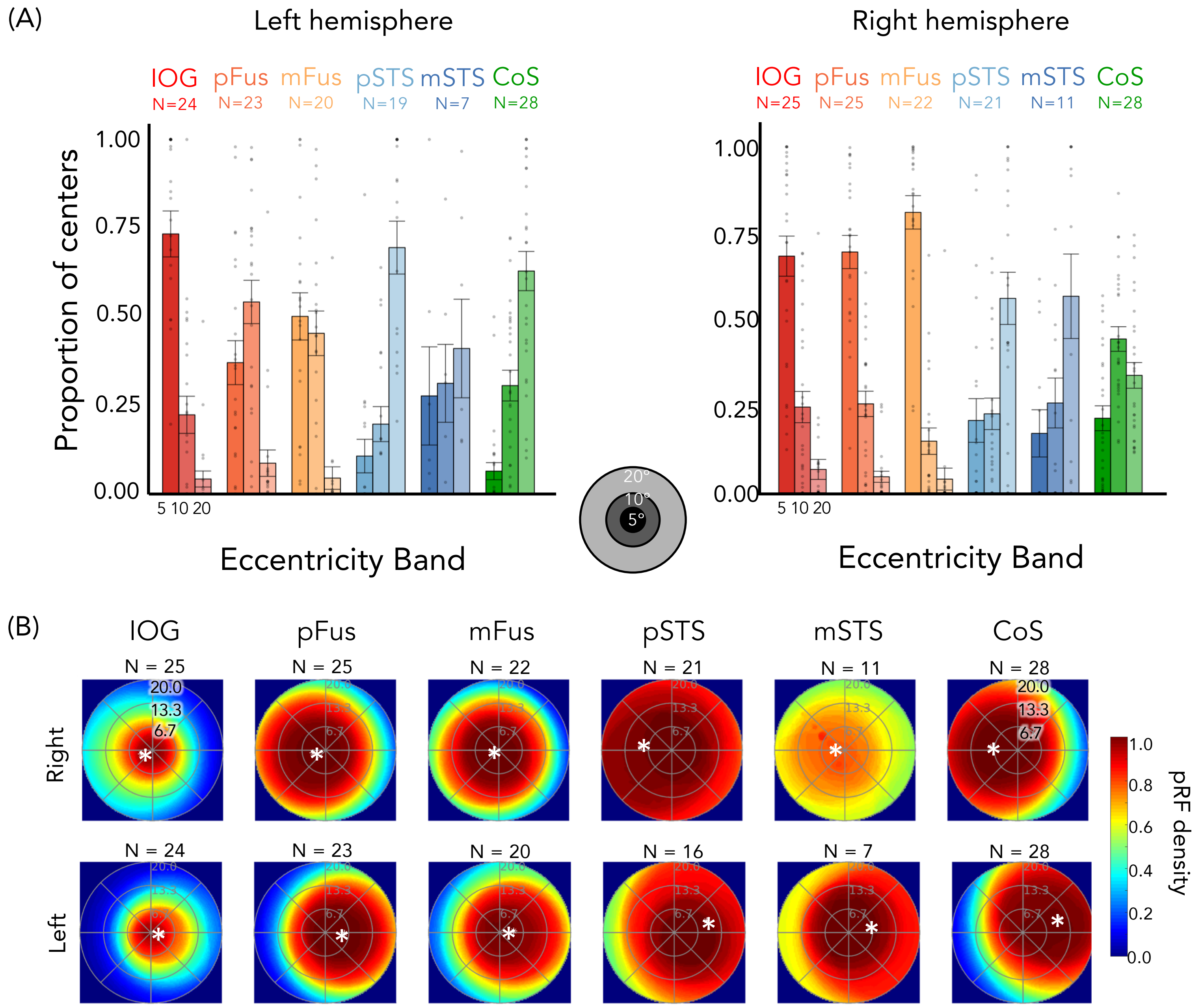

Supplementary Fig. 4: Control analysis limiting model fits to 20° of eccentricity. (A) Proportion of pRF centers of face-selective ROIs and CoS-places across eccentricities bands when model fits were constrained to the central 20°. For each participant and ROI, the proportion of pRF centers in each of three eccentricity bands (0°–5°; 5°–10°; 10°–20°) was calculated. As in the main results (figure 2B), a 2-way repeated-measures ANOVA on the proportion of centers for each hemisphere separately with factors of eccentricity band (0–5°/5–10°/10–20°/20–40°) and stream (ventral: IOG/pFus/mFus and lateral: pSTS/mSTS) confirms significant eccentricity band x stream interactions in both hemispheres (right: $F(2, 321) = 111.4$, $p < 2.2 \times 10^{-16}$; left: $F(2,276) = 58.9$, $p < 2.2 \times 10^{-16}$). *Bars:* mean across participants; Each dot is a participant; *Error bars*: ±SE. Corresponding main text figure: figure 2B. (B) Average visual field coverage of the central 20° for face-selective and CoS-places ROIs across participants. Corresponding main text figure 4A. Source data are provided as a Source Data file.

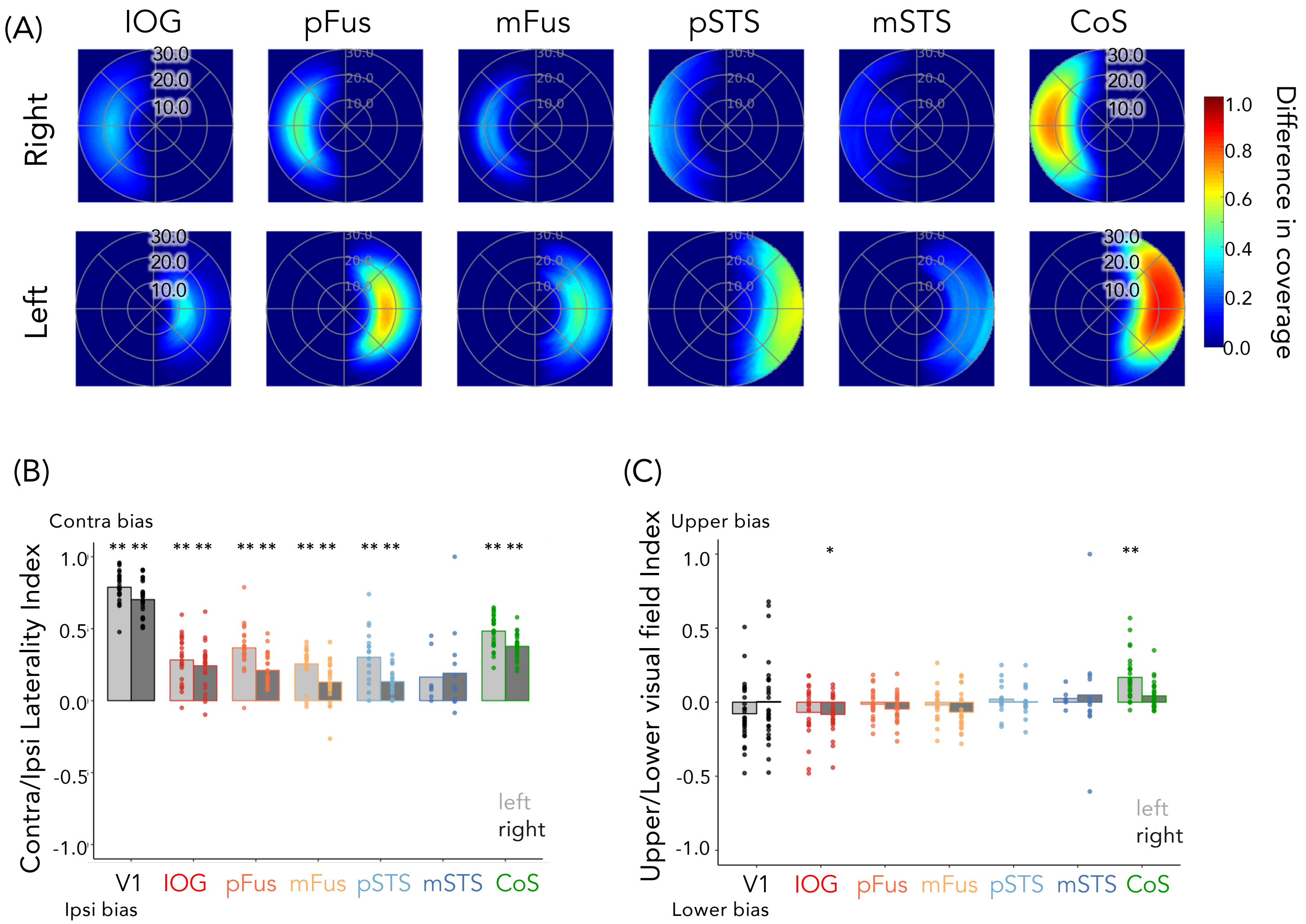

Supplementary Fig. 5: Contralateral preference, but not lower/upper visual field preference in face-selective regions. (A) Difference in the visual field coverage between the contralateral and ipsilateral visual fields. For each participant and ROI, the ipsilateral visual field was flipped across the vertical axis and subtracted from the contralateral visual field coverage. This difference in coverage was then averaged across participants. (B) Mean laterality index: $\frac{\text{contra} - \text{ipsi}}{\text{contra} + \text{ipsi}}$ of visual field coverage. Laterality of coverage was calculated for each participant and ROI. Asterisks reflect indices significantly than zero, two-sided t-tests, Bonferroni correction (* indicates p < .05; ** indicates p < .01). Left hemisphere - V1: $p=1.81\text{x}10^{-24}$, IOG: $p=1.74\text{x}10^{-7}$, pFus: $p=1.25\text{x}10^{-9}$, mFus: $p=3.08\text{x}10^{-7}$, pSTS: $p=2.05\text{x}10^{-5}$, mSTS: $p=.75$, CoS: $p=3.46\text{x}10^{-18}$. right hemisphere - V1: $p=9.43\text{x}10^{-21}$, IOG: $p=2.09\text{x}10^{-7}$, pFus: $p=7.45\text{x}10^{-9}$, mFus: $p=5.16\text{x}10^{-3}$, pSTS: $p=2.48\text{x}10^{-5}$, mSTS: $p=.47$, CoS: $p=1.26\text{x}10^{-18}$. (C) Mean Upper/lower index: $\frac{\text{upper} - \text{lower}}{\text{upper} + \text{lower}}$. Asterisks reflect indices significantly than zero, two-sided t-tests, Bonferroni correction (* indicates p < .05; ** indicates p < .01). Left hemisphere - V1: $p=.73$, IOG: $p=.96$, pFus: $p=.99$, mFus: $p=.99$, pSTS: $p=.99$, mSTS: $p=.99$, CoS: $p=2.87\text{x}10^{-5}$. right hemisphere - V1: $p=.99$, IOG: $p=.037$, pFus: $p=.37$, mFus: $p=.38$, pSTS: $p=.99$, mSTS: $p=.99$, CoS: $p=.30$. In (B, C) *Bars:* mean index across participants; *Dots:* individual participants' data; *Light bars:* left hemisphere; *Dark bars:* right hemisphere. For all panels: left hemisphere IOG-faces: N=24, pFus-faces: N=23, mFus-faces: N=21, pSTS-faces: N=19, mSTS-faces: N=7, CoS-places: N=28; right hemisphere IOG-faces: N=27, pFus-faces: N=25, mFus-faces: N=22, pSTS-faces: N=21, mSTS-faces: N=13, CoS-places: N=28. Source data are provided as a Source Data file.

(A) IOG

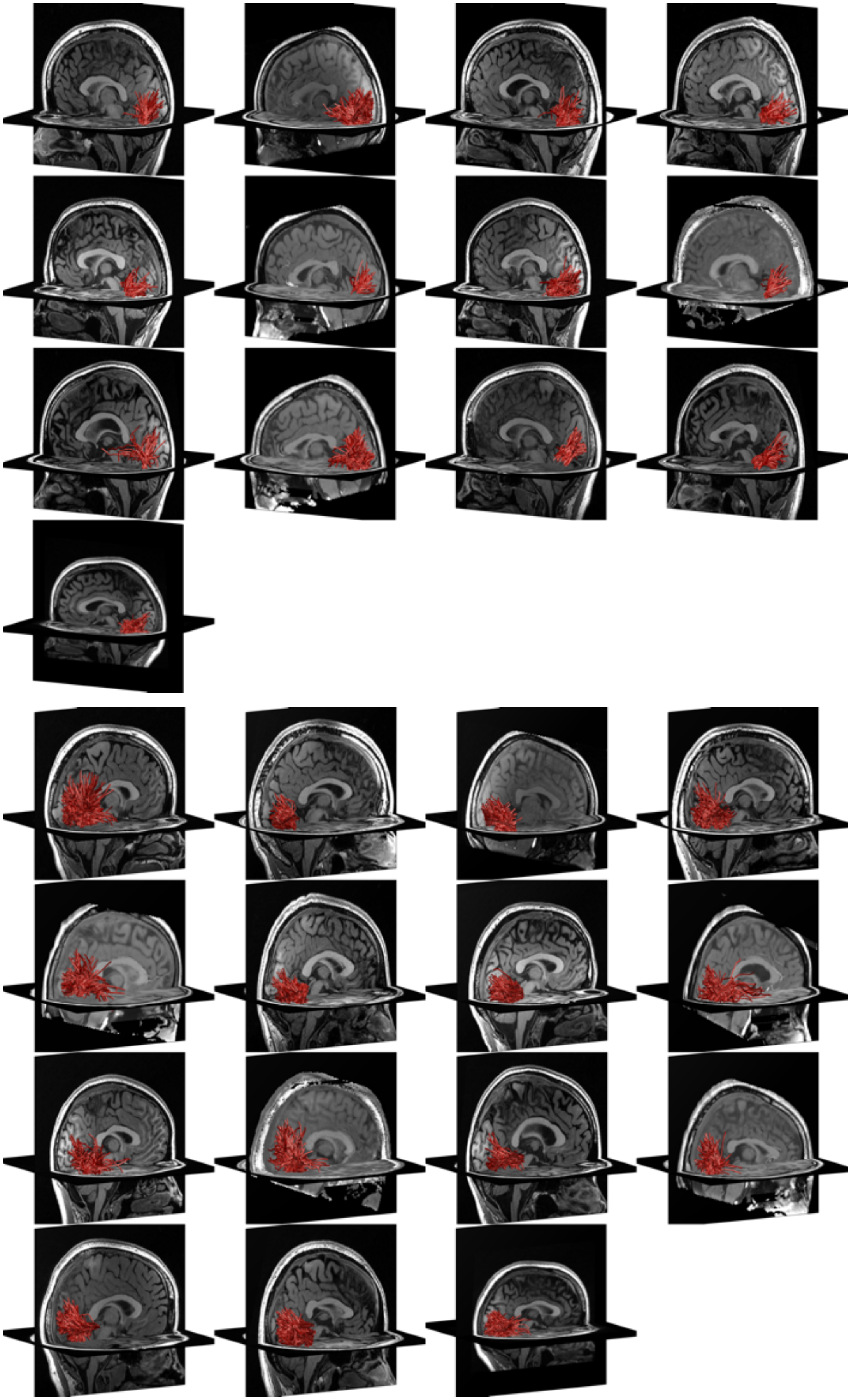

(B) pFus

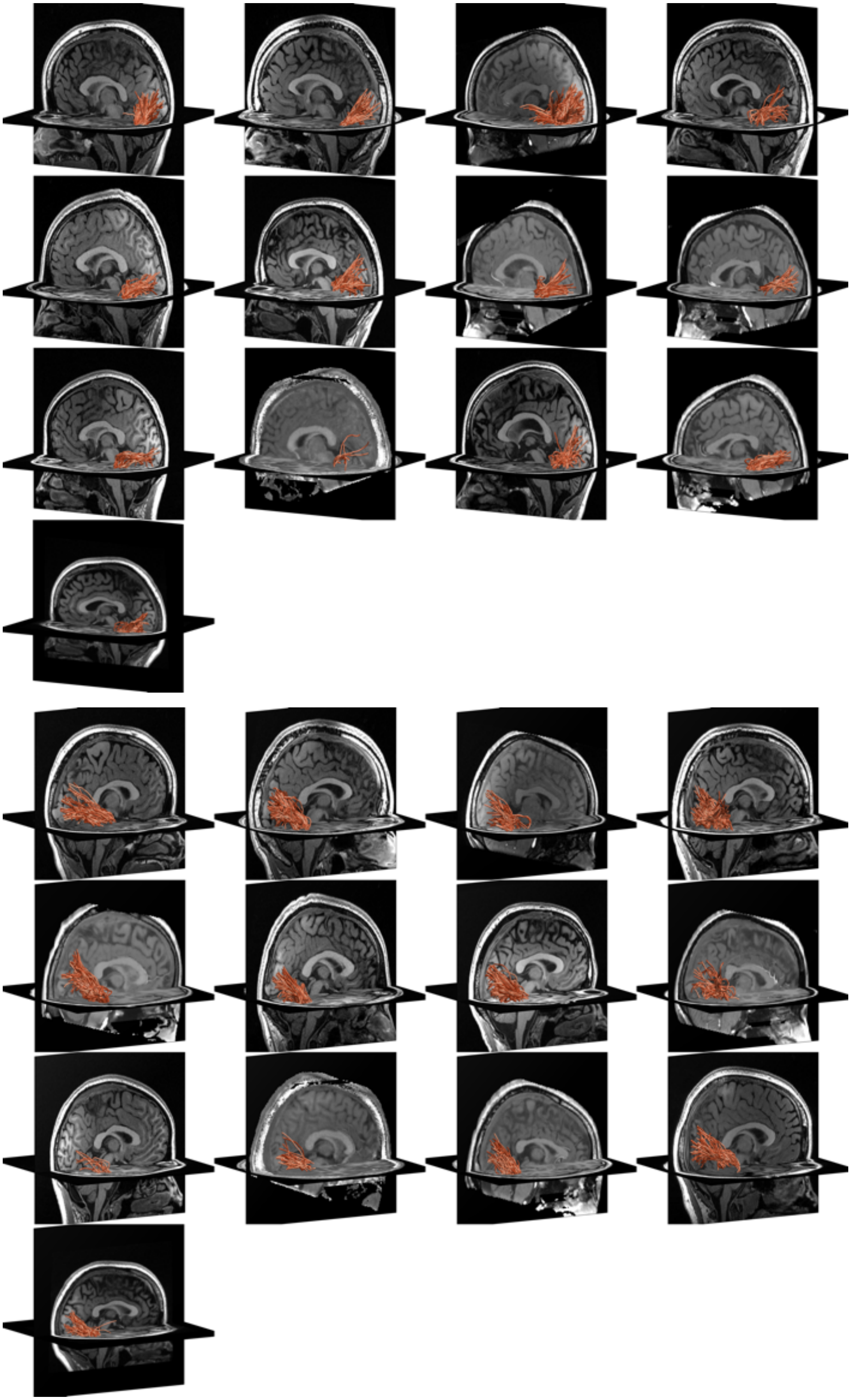

(C) mFus-faces

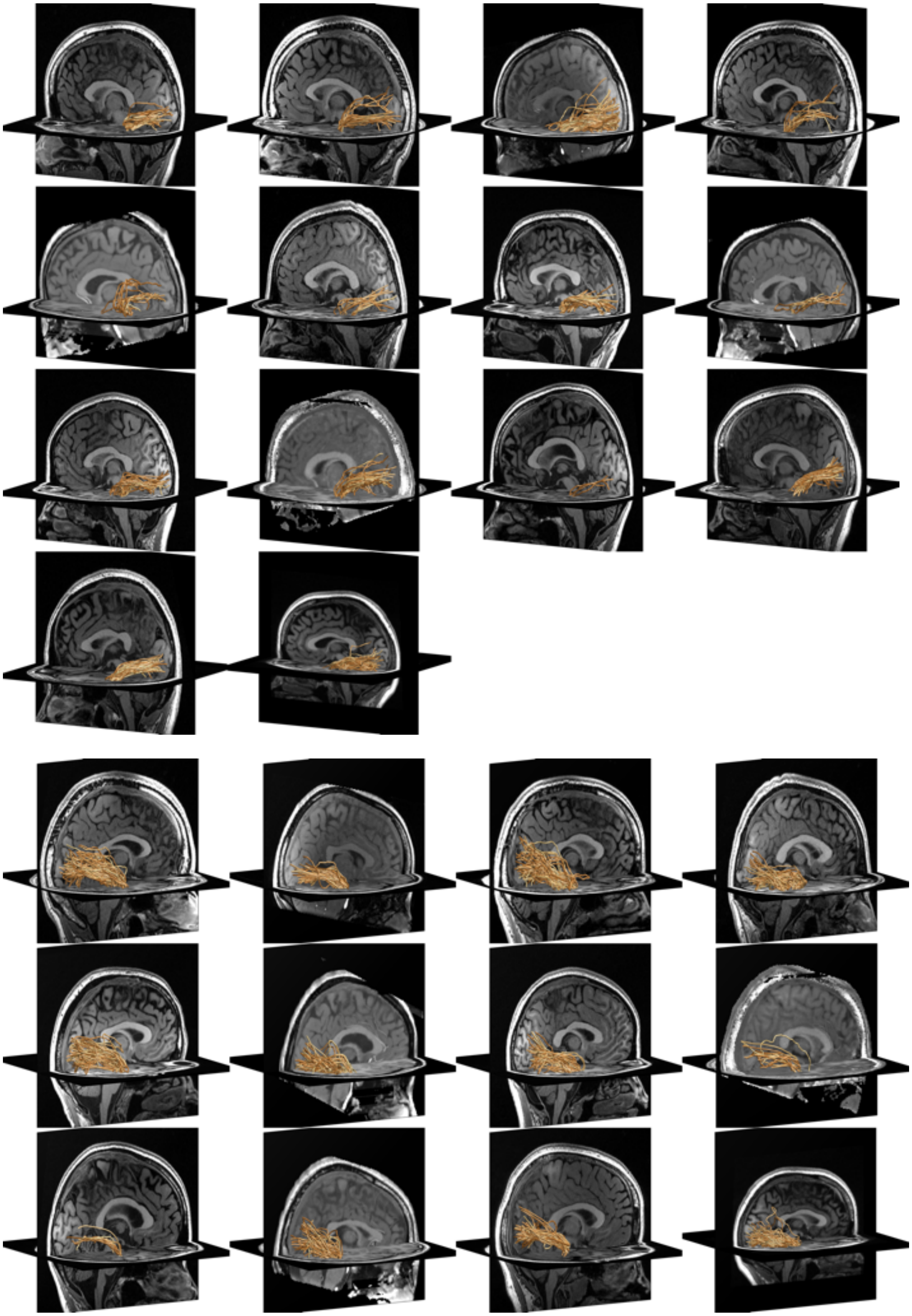

(D) pSTS-faces

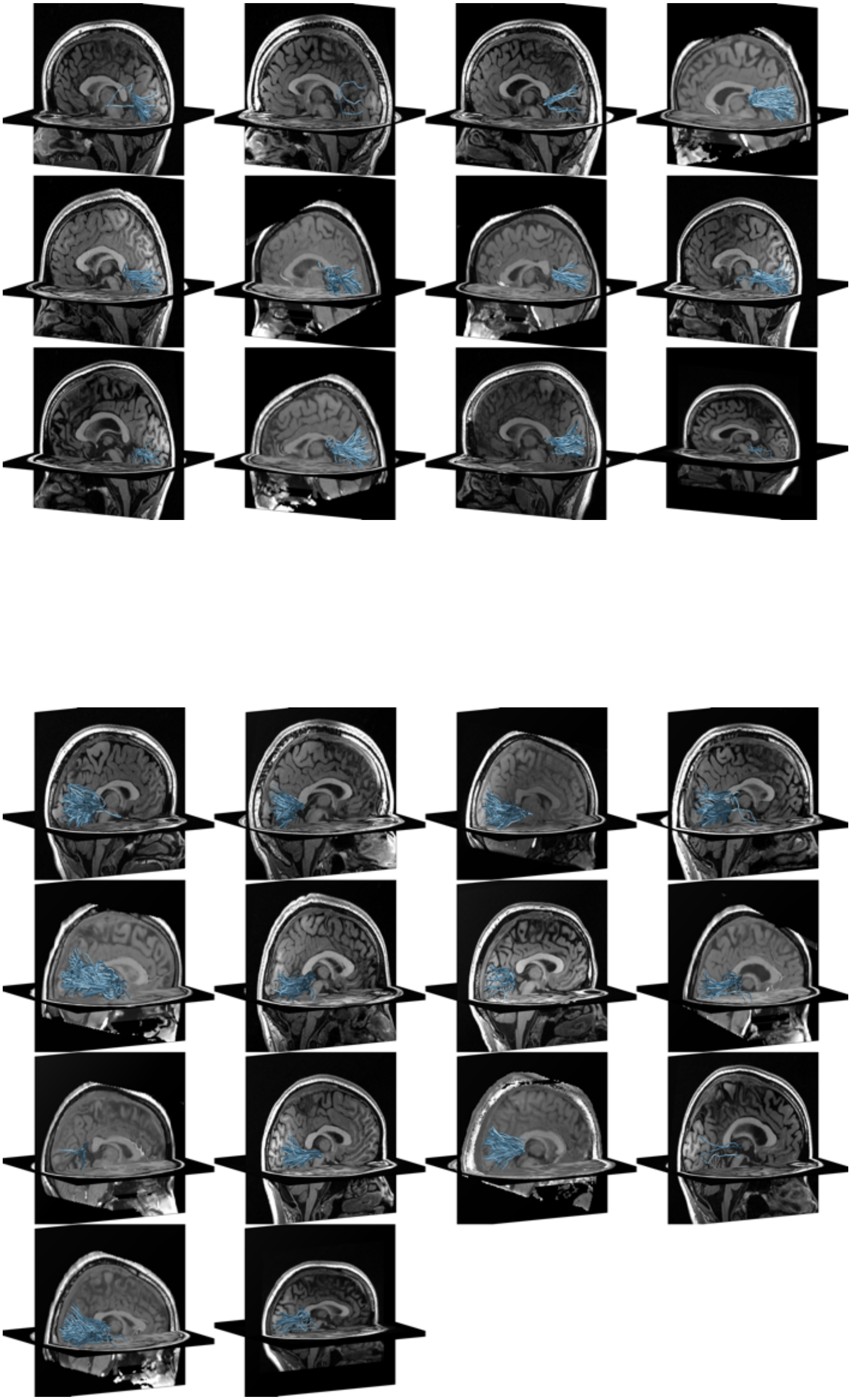

(E) mSTS-faces

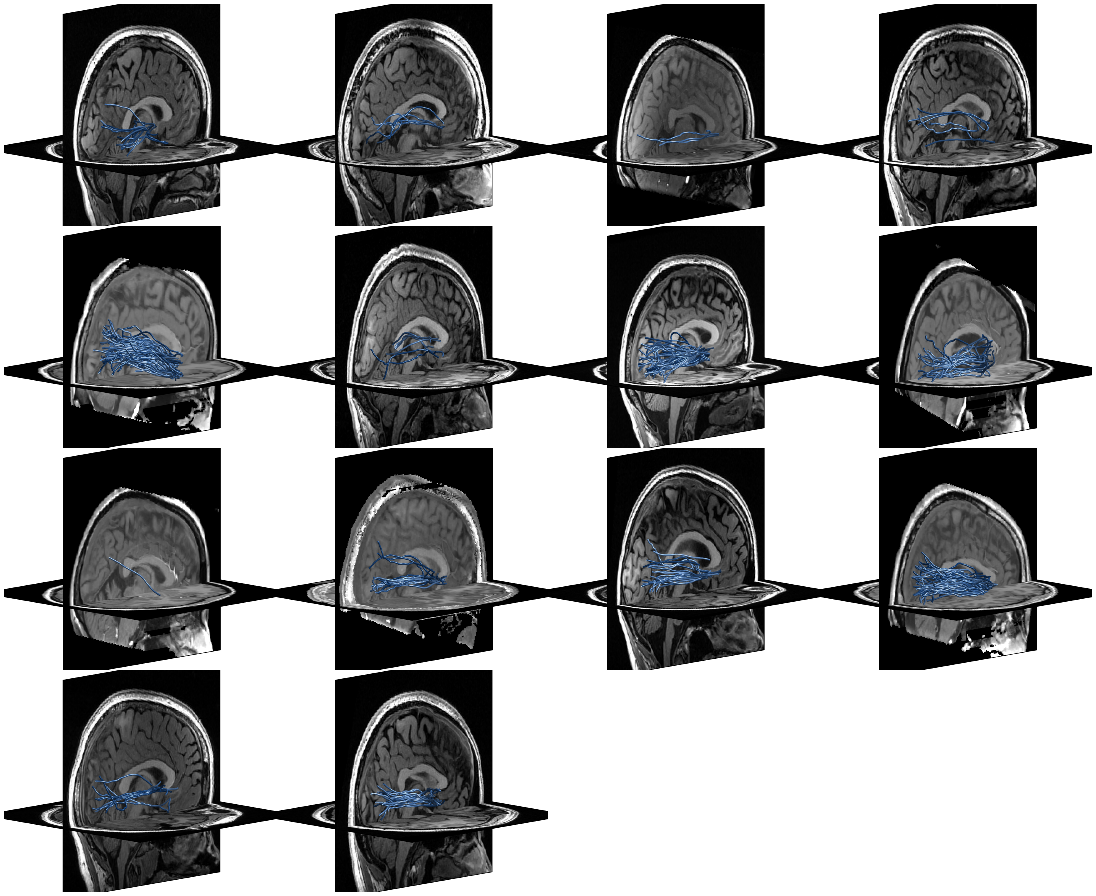

(F) CoS-places

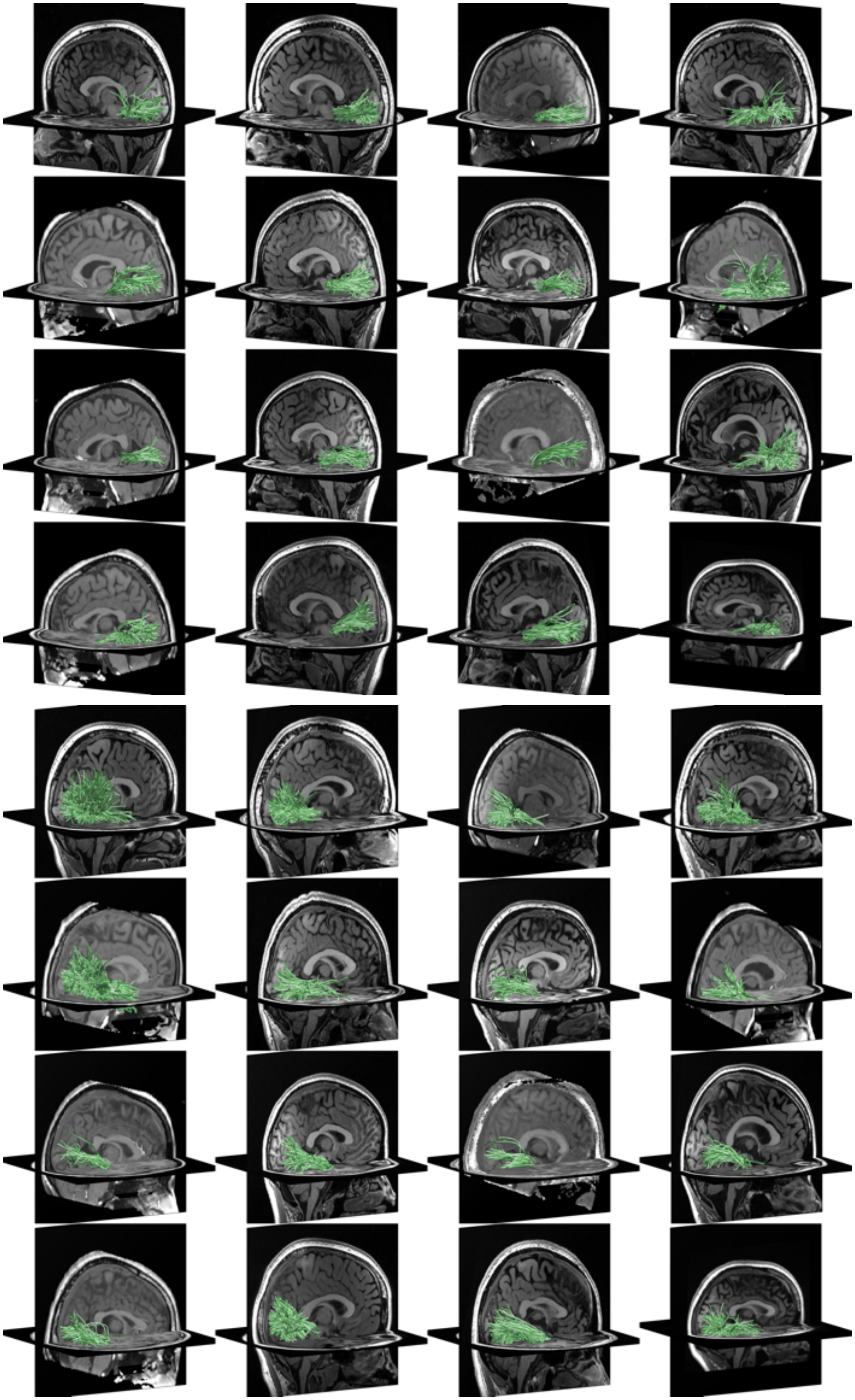

Supplementary Fig. 6: Functionally defined white-matter tracts (fDWT) between early visual cortex and high-level visual regions were defined as all tracts that intersect both EVC and the functional ROI. All analyses were done within each participant's brain. Each panel is participant; *Top brains:* left hemisphere; *Bottom brains:* right hemisphere. (A) IOG-faces, (B), pFus-faces, (C) mFus-faces, (D) pSTS-faces, (E) mSTS faces, and (F) CoS-places. Corresponding main text figure: figure 6A.

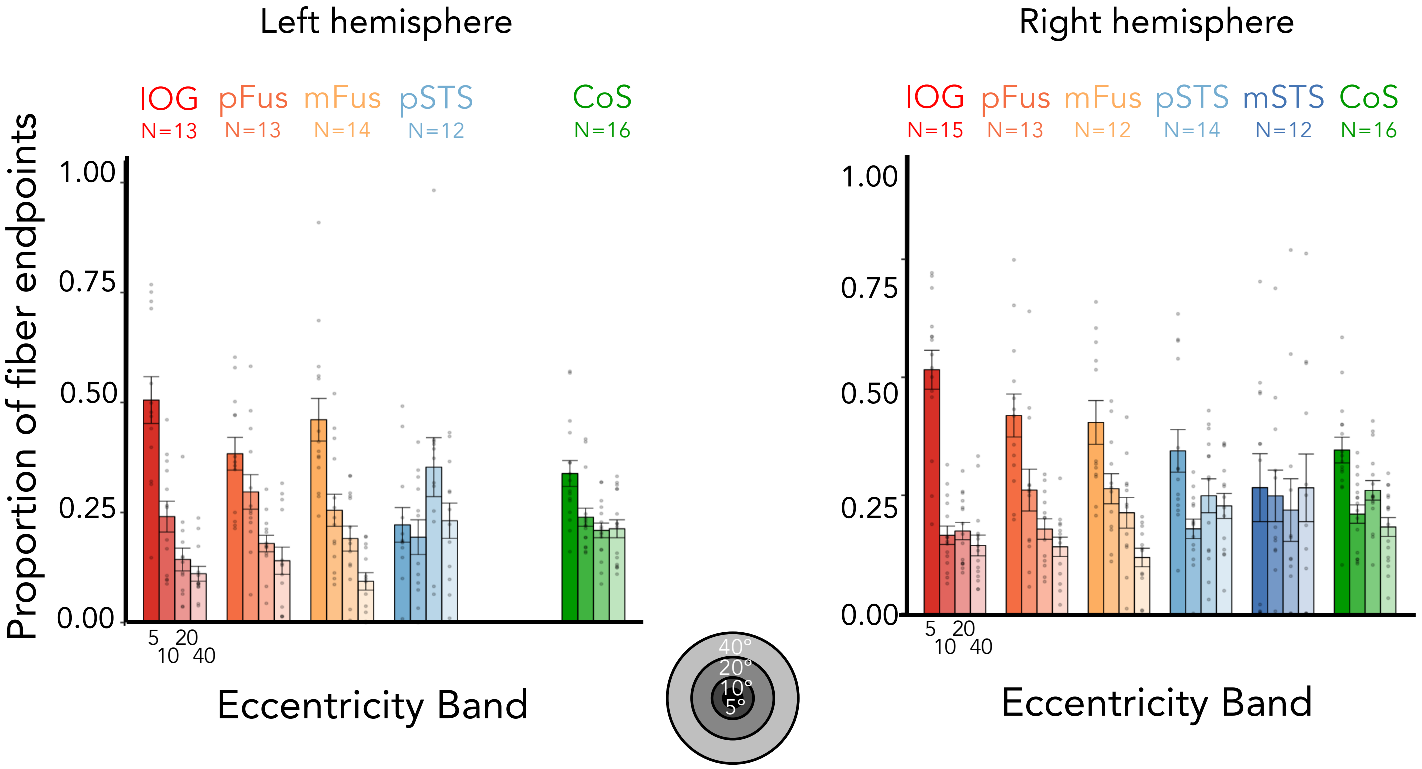

Supplementary Fig. 7: 5mm disk ROI control. The average proportion of fiber endpoints from each face-selective region (and CoS-places) that terminate in each of four eccentricity bands (0°–5°; 5°–10°; 10°–20°; 20°–40°) in EVC, determined using individual participant retinotopy in each participant's native brain space. Functional ROIs were determined by creating 5mm disk ROIs at the center of each functionally defined ROI from the localizer task in order to control for ROI size. As in the main results (figure 8B), a 2-way repeated-measures ANOVA on the proportion of fiber endpoints in EVC for each hemisphere separately with factors of eccentricity band (0–5°/5–10°/10–20°/20–40°) and stream (ventral: IOG/pFus/mFus and lateral: pSTS/mSTS; mSTS right hemisphere only) confirms significant eccentricity band x stream interactions in both hemispheres (right: $F(3, 256) = 8.7$, $p = 1.65 \times 10^{-5}$; left: $F(3,200) = 17.3$, $p = 5.248 \times 10^{-10}$). *Bars:* mean across participants; Each dot is a participant; *Error bars*: ±SE. Corresponding main text figure: figure 7B. Source data are provided as a Source Data file.

mSTS-faces ROI comparison

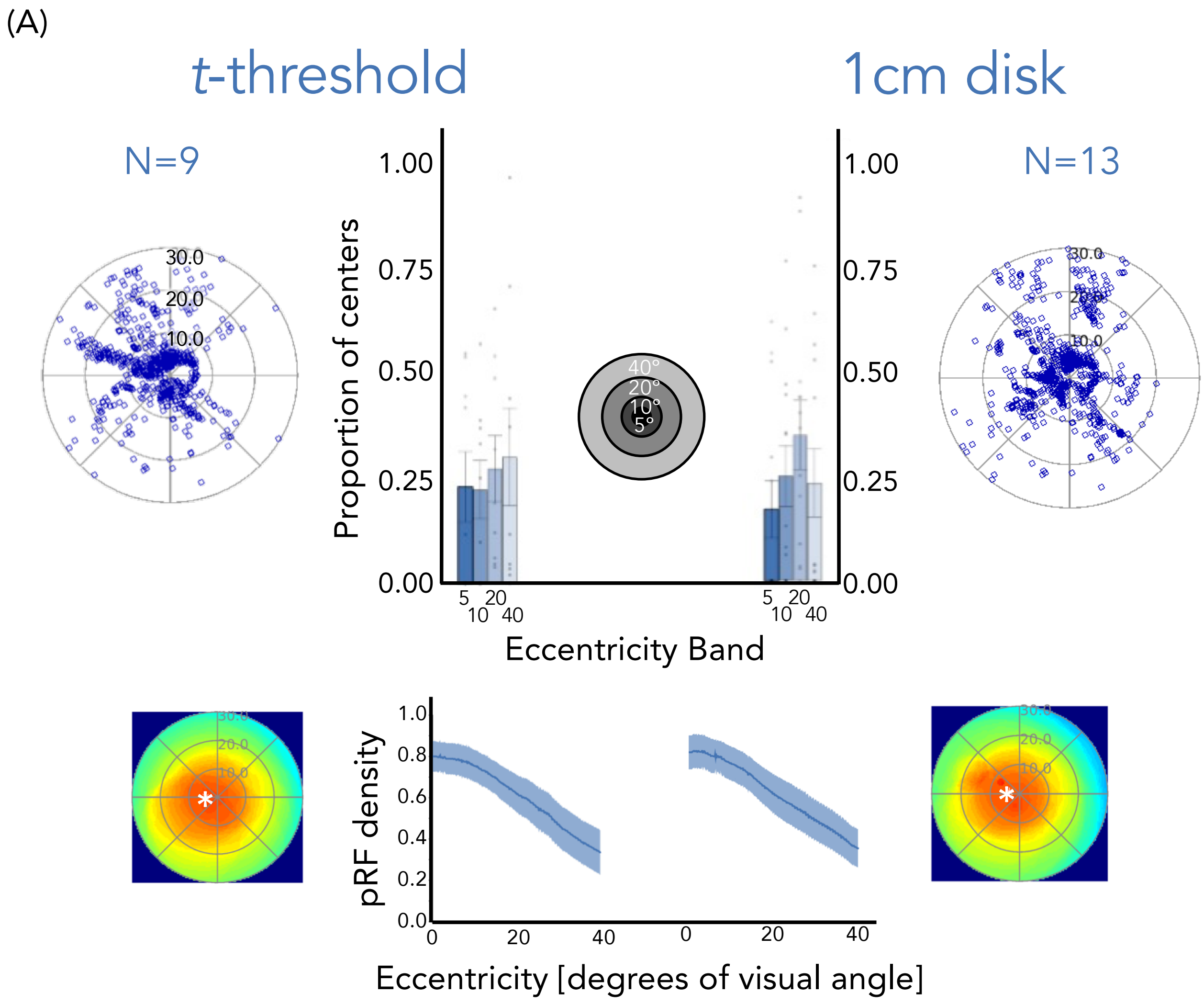

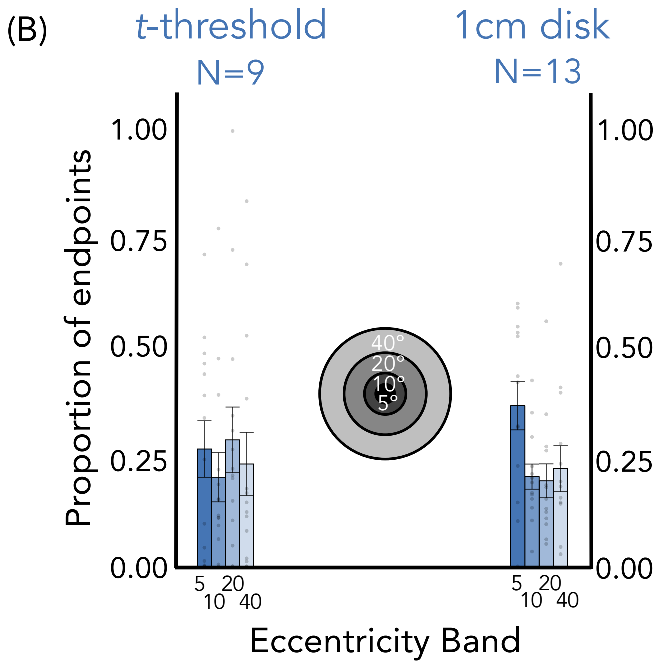

Supplementary Fig. 8: Comparison of right mSTS-faces ROIs. To ensure that we captured the full extent of mSTS-faces despite using a static functional localizer, we created 1cm disk ROIs centered on each participant's functional ROI. However, pRF results were highly similar regardless of which ROI (disk or based purely on functional threshold of t>3) as used (panel A). The same was true for the proportion of fiber endpoints analysis (panel B); though, if anything, the effect was slightly more pronounced in the *t*-threshold ROI vs. the disk ROI. For bar plots, dots represent individual participant values and error bars ±SE. For the pRF density across eccentricity plot, shaded area represents ±SE.

Supplementary Table 1. Post-hoc Tukey tests for repeated measures ANOVA on pRF size (factor: ROI), significant contrasts in bold

Right hemisphere:

contrast	***df***	***t ratio***	***p-value***
IOG - mFus	81.12	-0.86	0.91
IOG - mSTS	**90.58**	**-4.52**	**0.0002**
IOG - pFus	80.20	-1.19	0.75
IOG - pSTS	**82.80**	**-6.11**	**3.13e-07**
mFus - mSTS	**91.33**	**-3.66**	**0.0038**
mFus - pFus	84.69	-0.29	0.99
mFus - pSTS	**86.06**	**-5.00**	**2.91e-05**
mSTS - pFus	**94.76**	**3.550**	**0.0064**
mSTS - pSTS	93.30	-0.65	0.97
pFus - pSTS	**81.35**	**-4.87**	**0.0001**

Left hemisphere:

contrast	***df***	***t ratio***	***p-value***
IOG - mFus	72.45	-2.47	0.11
IOG - mSTS	**77.74**	**-6.37**	**1.20e-07**
IOG - pFus	69.67	-2.45	0.12
IOG - pSTS	**71.12**	**-7.96**	**1.22e-10**
mFus - mSTS	**75.54**	**-4.56**	**0.0002**
mFus - pFus	74.08	0.099	0.99
mFus - pSTS	**74.85**	**-5.28**	**1.18e-05**
mSTS - pFus	**79.05**	**4.63**	**0.0001**
mSTS - pSTS	79.15	0.60	0.97
pFus - pSTS	**71.66**	**-5.55**	**4.49e-056**

Supplementary Table 2. Post-hoc Tukey tests for repeated measures ANOVA on pRF size within eccentricity band (factor: ROI) in the right hemisphere, significant contrasts in bold

0 to 5°:

contrast	*df*	*t ratio*	*p-value*
IOG - mFus	87	-1.30	0.69
IOG - mSTS	87	-0.42	0.99
IOG - pFus	87	-1.60	0.51
IOG - pSTS	**87**	**-6.67**	**2.31-08**
mFus - mSTS	87	0.34	0.99
mFus - pFus	87	-0.25	0.99
mFus - pSTS	**87**	**-5.37**	**6.26e-06**
mSTS - pFus	87	-0.49	0.99
mSTS - pSTS	**87**	**-3.89**	**0.0018**
pFus - pSTS	**87**	**-5.29**	**8.89e-06**

5 to 10°:

contrast	*df*	*t ratio*	*p-value*
IOG - mFus	68.47	-0.27	0.99
IOG - mSTS	**80.14**	**-3.00**	**0.029**
IOG - pFus	66.71	-0.60	0.97
IOG - pSTS	**69.70**	**-3.94**	**0.0017**
mFus - mSTS	80.78	-2.73	0.0576
mFus - pFus	69.99	-0.31	0.99
mFus - pSTS	**71.56**	**-3.56**	**0.0058**
mSTS - pFus	83.28	2.58	0.0828
mSTS - pSTS	82.73	-0.11	0.99
pFus - pSTS	**67.34**	**-3.45**	**0.0085**

Supplementary Table 3. Post-hoc Tukey tests for repeated measures ANOVA on slopes of pRF density by eccentricity lines (factor: ROI), significant contrasts in bold

Right hemisphere:

contrast	***df***	***t ratio***	***p-value***
IOG - mFus	**80.93**	**4.11**	**0.0009**
IOG - mSTS	**87.98**	**-4.73**	**0.0001**
IOG - pFus	**81.46**	**4.17**	**0.0007**
IOG - pSTS	**83.45**	**-7.11**	**3.80e-09**
mFus - mSTS	**87.42**	**-7.96**	**3.89e-10**
mFus - pFus	84.04	-0.078	0.99
mFus - pSTS	**84.92**	**-10.73**	**2.04e-10**
mSTS - pFus	**90.27**	**8.02**	**4.80e-10**
mSTS - pSTS	89.14	-1.22	0.74
pFus - pSTS	**81.47**	**-11.06**	**4.34e-12**

Left hemisphere:

contrast	***df***	***t ratio***	***p-value***
IOG - mFus	80.95	2.74	0.057
IOG - mSTS	**88.30**	**-4.12**	**0.0008**
IOG - pFus	**78.71**	**3.32**	**0.012**
IOG - pSTS	**80.95**	**-5.06**	**2.50e-05**
mFus - mSTS	**89.53**	**-6.31**	**1.02e-07**
mFus - pFus	83.07	0.46	0.99
mFus - pSTS	**84.29**	**-7.47**	**8.52e-10**
mSTS - pFus	**87.96**	**6.88**	**8.55e-09**
mSTS - pSTS	88.19	-0.32	0.99
pFus - pSTS	**81.75**	**-8.18**	**8.61e-11**

Supplementary Notes: Validating key results with a threshold of 10% variance explained (voxel level) by pRF model

As a further test of our results, we have reexamined the data with a more lenient threshold of 10% variance explained by pRF model per voxel. This allows inclusion of more data as we include ROIs with at least 10 voxels which exceed this variance explained threshold. All the key effects replicate with this threshold.

<u>Difference in proportion centers across streams (related to pg. 8):</u> A 2-way repeated measures LMM ANOVA on the proportion of centers with eccentricity band (0–5°/5–10°/10–20°/20–40°) and stream (ventral: IOG/pFus/mFus and lateral: pSTS/mSTS) as factors revealed a significant eccentricity band x stream interaction in both hemispheres (right: $F(3, 460)=101.0$, $p<2.2x10^{-16}$; left: $F(3,384)=56.7$, $p=2.2x10^{-16}$). Post-hoc Tukey's tests establish that this is driven by a significantly higher proportion of centers in the most foveal 0–5° eccentricity band in ventral vs. lateral face-selective regions (proportion higher in ventral than lateral 0–5°: right: 0.56±0.04, $t(460)=13.9$, $p<.0001$; left: 0.43±0.05, $t(384)=8.5$, $p<.0001$), as well as a significantly lower proportion of centers for ventral vs. lateral regions in the two most peripheral eccentricity bands (proportion lower in ventral than lateral, 10–20° right: 0.27±0.04, $t(460)=-6.8$, $p<.0001$; left: 0.28±0.05, $t(384)=-5.6$, $p<.0001$; 20–40° right: 0.32±0.04, $t(460)=-7.9$, $p<.0001$; left: 0.36±0.05, $t(384)=-7.1$ $p<.0001$).

<u>Differences in pRF size across streams (related to pg. 9):</u> Results show that in both hemispheres pRFs were significantly larger in lateral than ventral face-selective regions (paired t-tests; right: $t(26)=-3.9$, $p=.00057$; left: $t(23)=-5.5$, $p=1.2x10^{-5}$). Differences between ROIs were significant (1-way repeated measures LMM ANOVAs on median pRF size, right ROIs: IOG/pFus/mFus/pSTS/mSTS, $F(4,93)=23.2$, $p<2.6x10^{-13}$; left ROIs: IOG/pFus/mFus/pSTS/mSTS, $F(4,76)=15.6$, $p=2.4x10^{-9}$), and were driven by significant differences between both pSTS-faces and each of the ventral face-selective regions (post-hoc Tukey tests, all ts>5.0, ps<.0001).

<u>Difference in visual field coverage across streams (related to pg. 11):</u> Results reveal (i) significant differences between the average slopes of ventral and lateral face-selective regions (paired t-tests; right: $t(25)=-10.5$, $p=1.1x10^{-10}$; left: $t(25)=-10.2$, $p=2.4x10^{-10}$), whereby slopes for ventral face-selective ROIs were more negative than for lateral face-selective ROIs and (ii) significant differences between the average slopes of individual face-selective ROIs (right: $F(4,92)=53.0$, $p<2.2x10^{-16}$; left: $F(4,85)=34.3$, $p<2.2x10^{-13}$, 1-way repeated measures LMM ANOVAs on the slopes with factor ROI). Specifically, slopes in lateral face-selective regions—pSTS-faces and mSTS-faces—were significantly closer to zero than any of the ventral face-selective regions (all ts>4.7, ps<=.0001, post-hoc Tukey tests). Additionally, bilateral pFus-faces and right mFus-faces had significantly more negative slopes than IOG-faces (all ts<-3.3, ps<=.0098), indicating that the former ROIs have a larger foveal bias than the latter. Similarly, the parameters for both the inflection point and the lower asymptote in the generalized logistic function are significantly different between ventral and lateral regions in both hemispheres, such that ventral face-selective regions have smaller valued lower asymptotes (paired t-tests; right: $t(25)=-3.6$,

p=0.0015; left: t(23)=-4.9, $p=5.4x10^{-5}$) and inflection points (paired t-tests; right: t(25)=-3.2 p=0.0030; left: t(23)=-3.6, p=0.0015) than lateral face-selective regions.
